# Supplementary material for: Identification and Characterization of CYP6 Family Genes from the Oriental Fruit Moth (Grapholita molesta) and Their Responses to Insecticides
Source: Insects. 2022 Mar 17;13(3):300. doi: 10.3390/insects13030300 (PMC8953268; doi:10.3390/insects13030300)
Supplement: Supplementary file 1 [file insects-13-00300-s001.zip › File S1.pptx]

## Slide 1
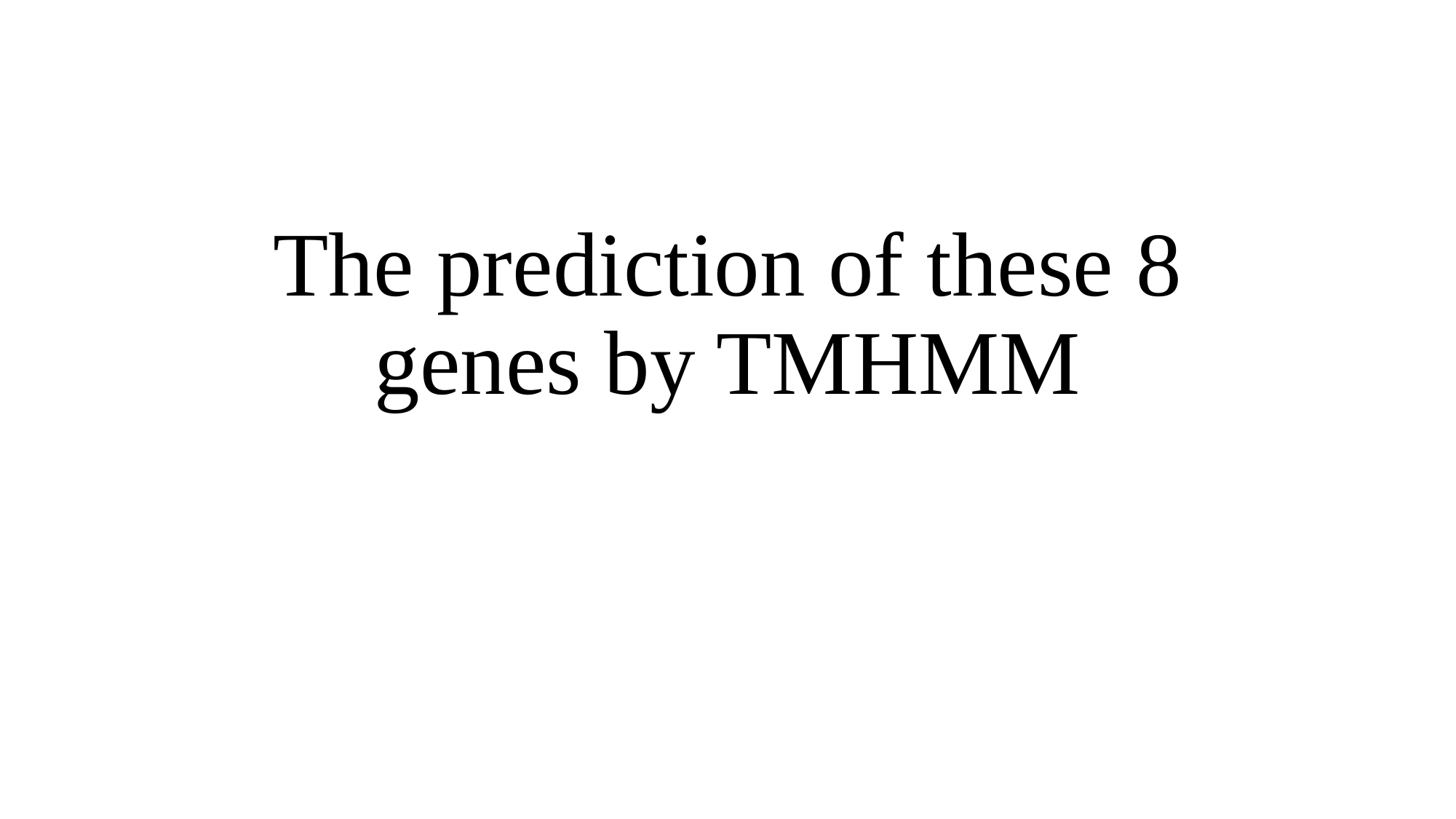

# The prediction of these 8 genes by TMHMM

## Slide 2
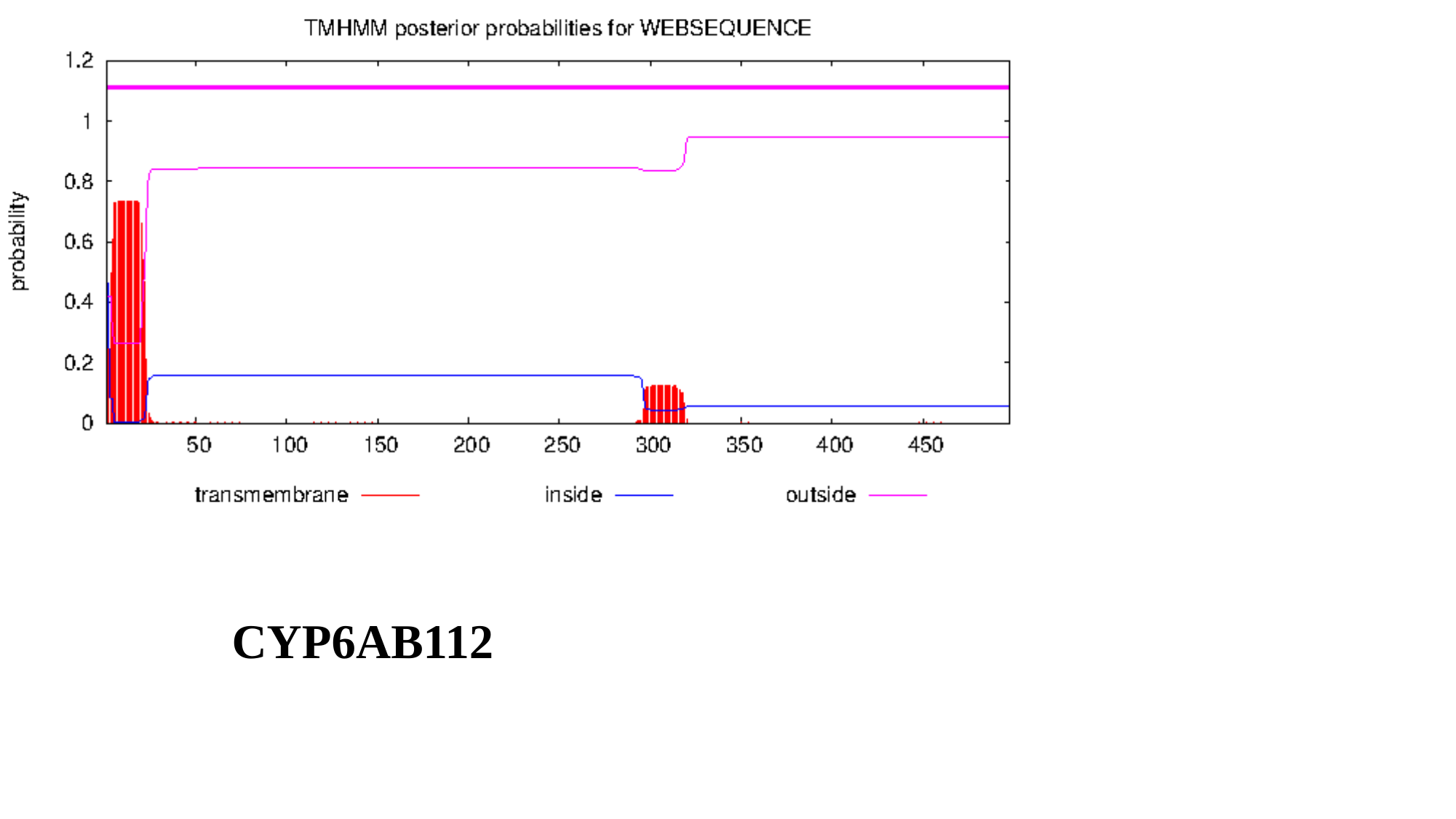

CYP6AB112

## Slide 3
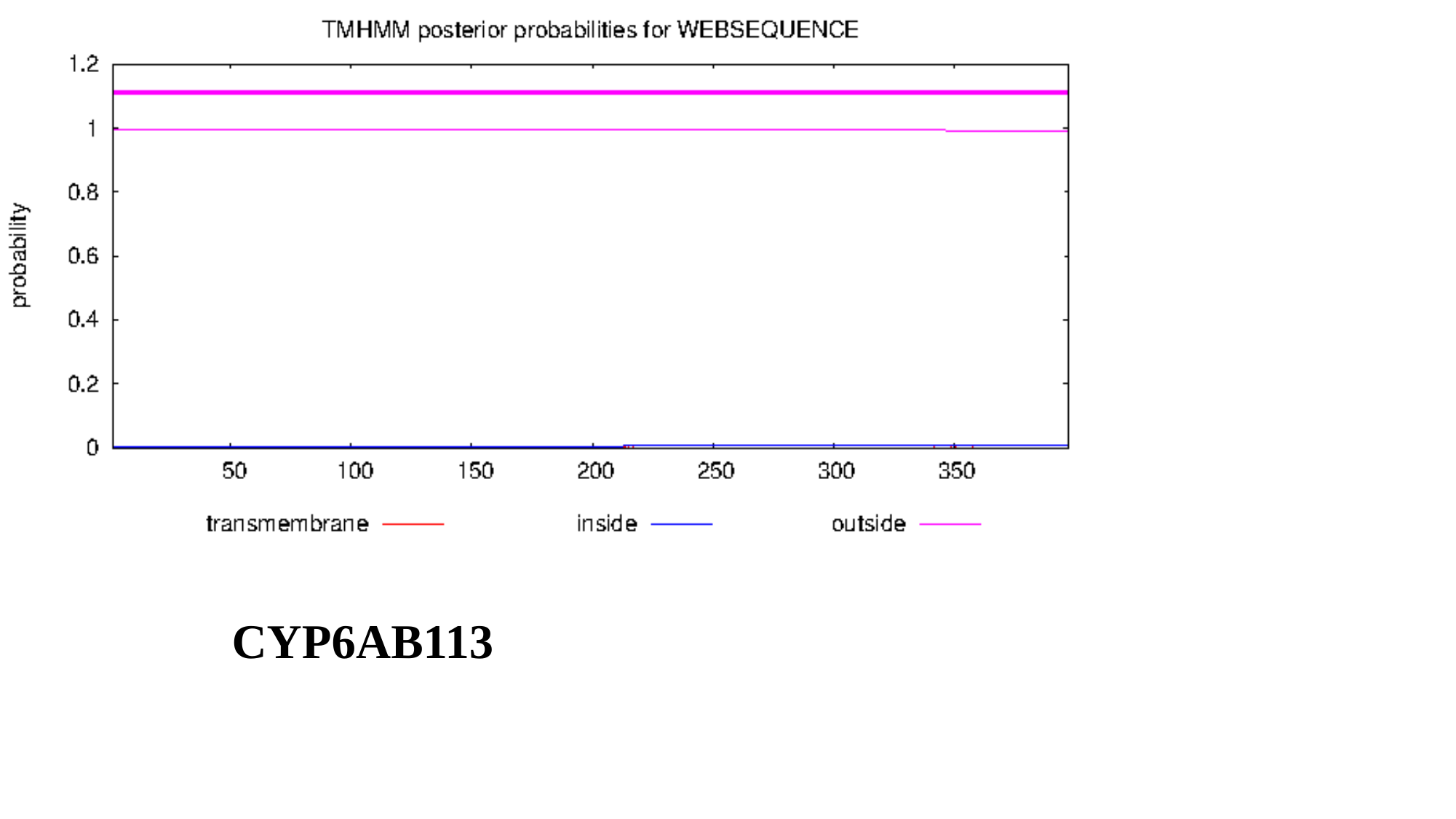

CYP6AB113

## Slide 4
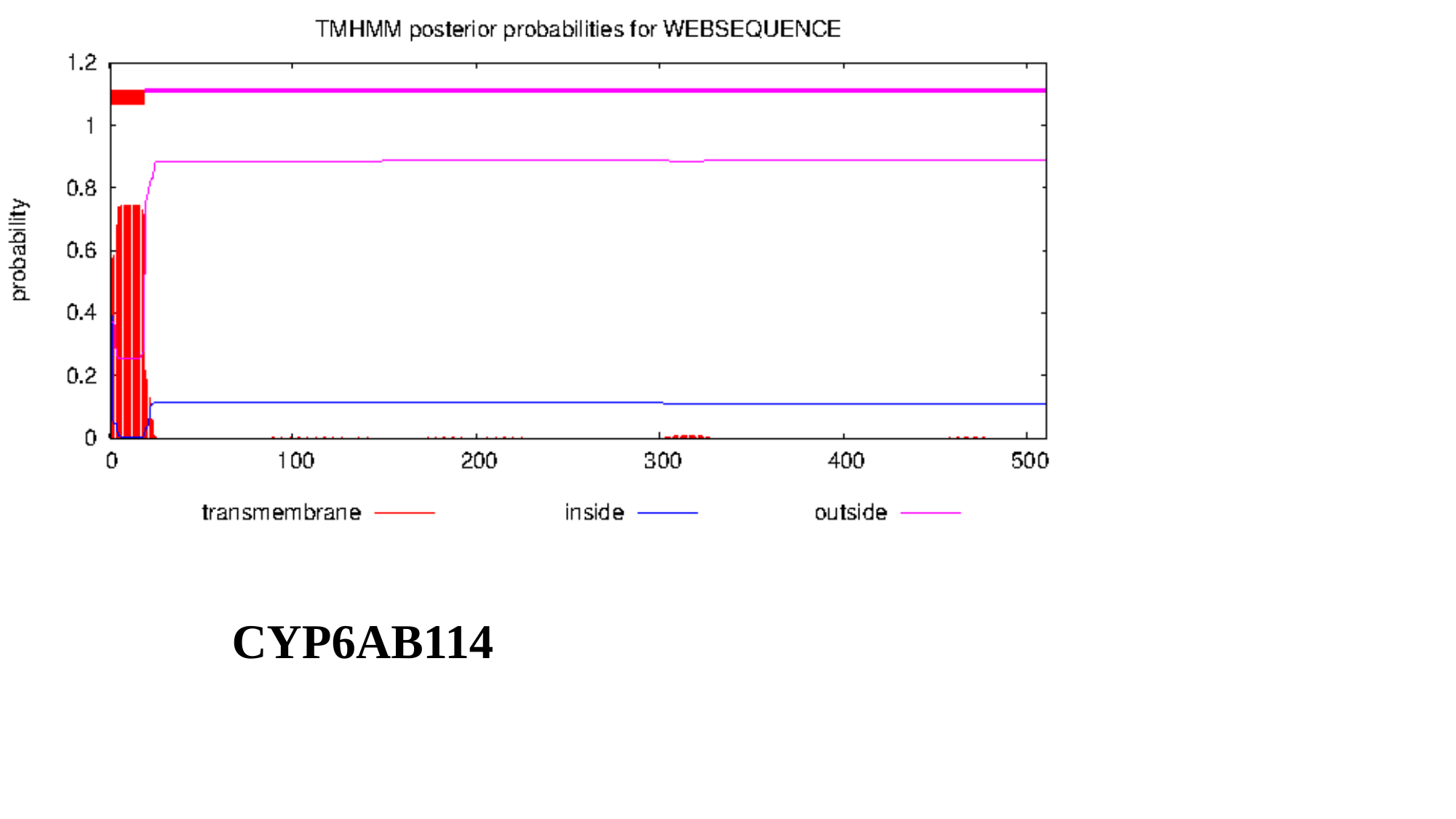

CYP6AB114

## Slide 5
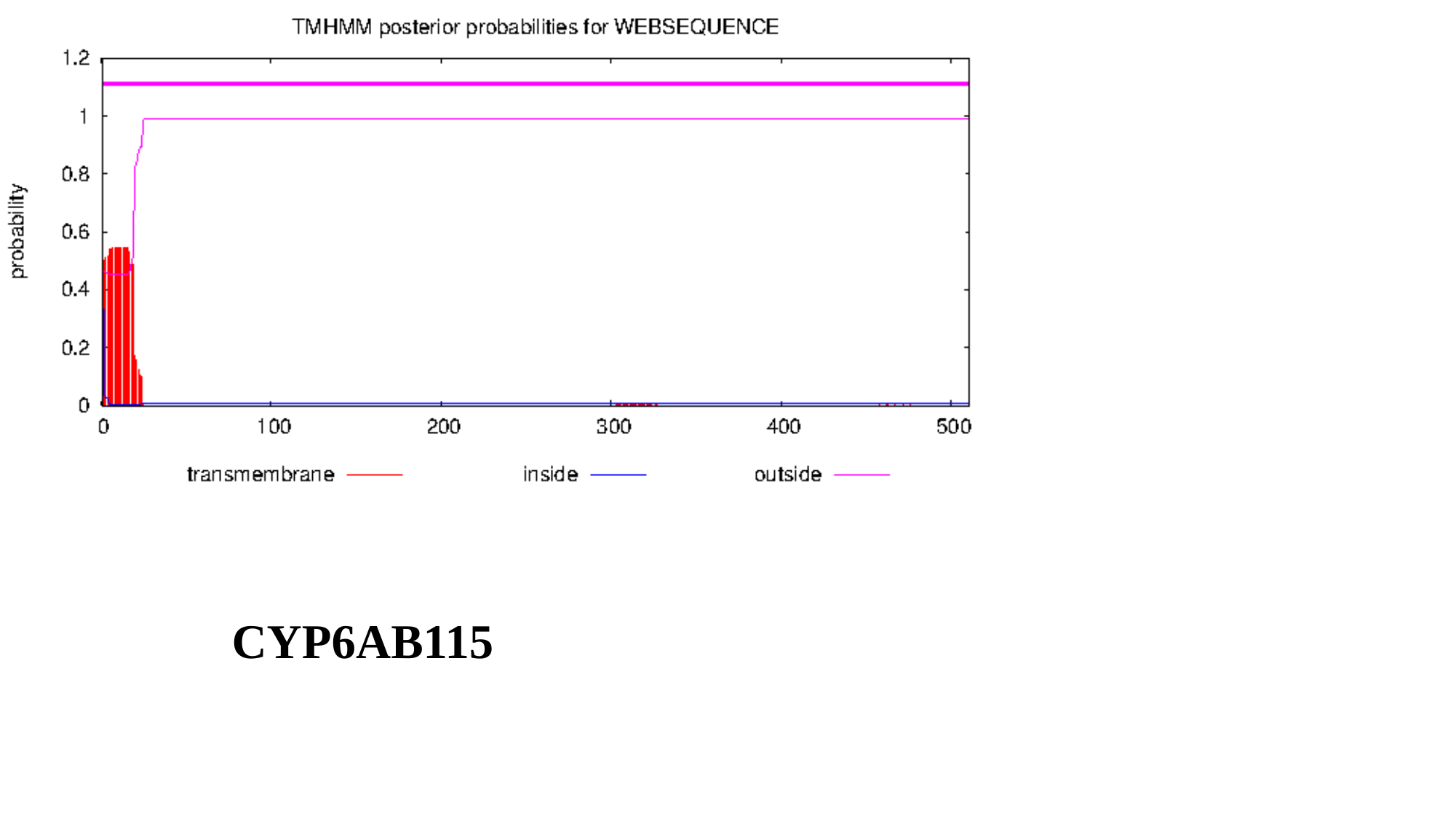

CYP6AB115

## Slide 6
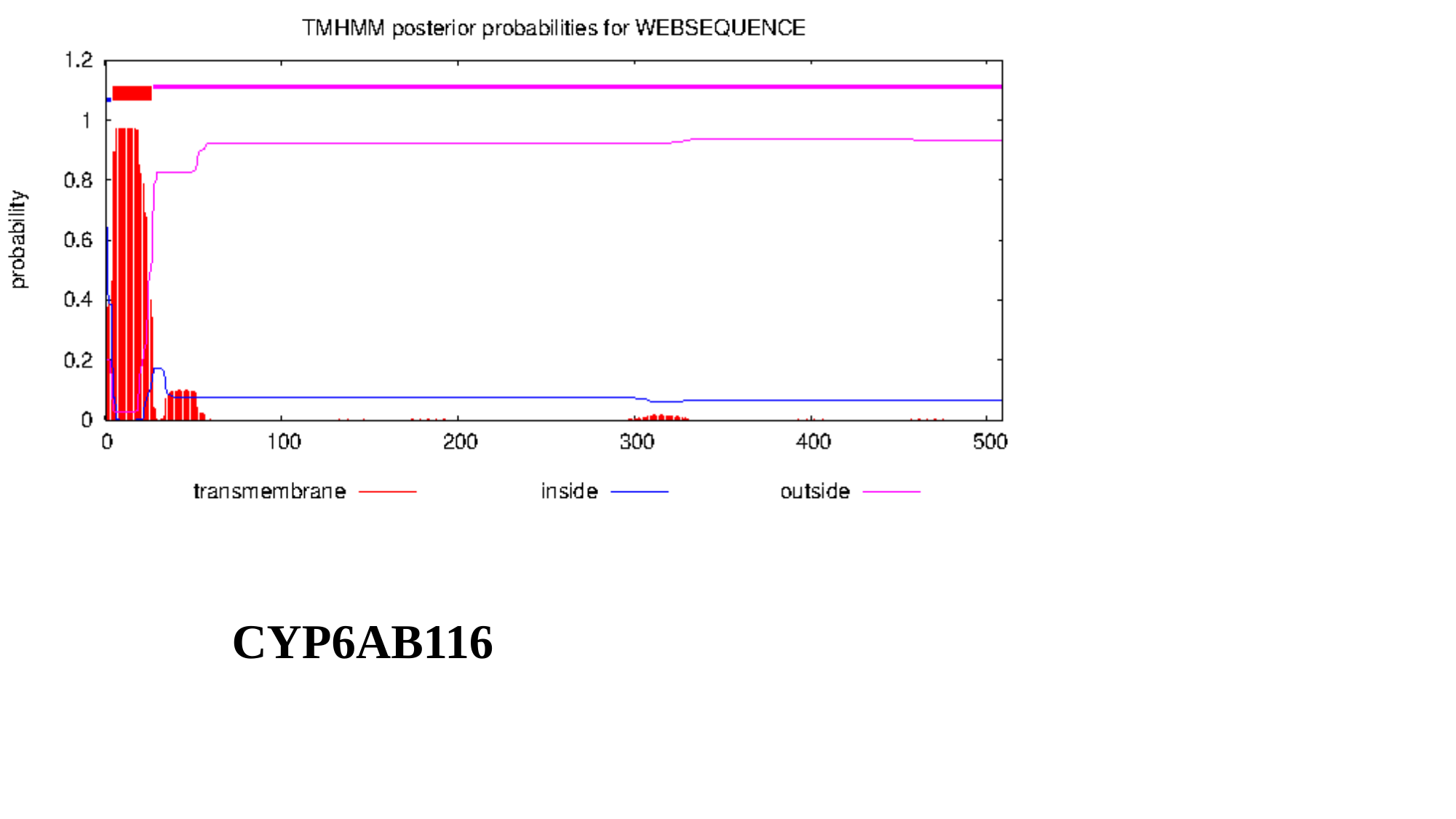

CYP6AB116

## Slide 7
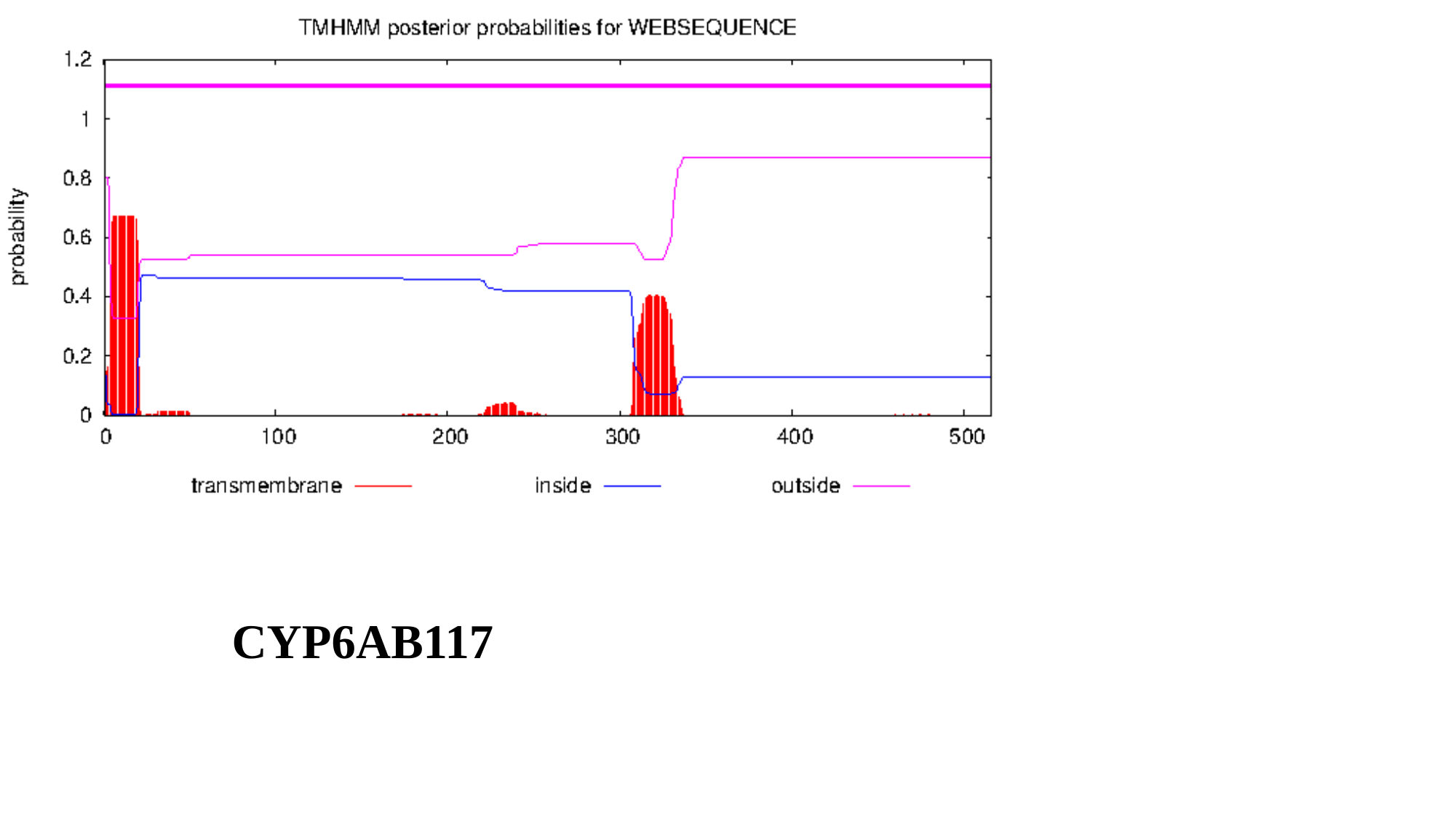

CYP6AB117

## Slide 8
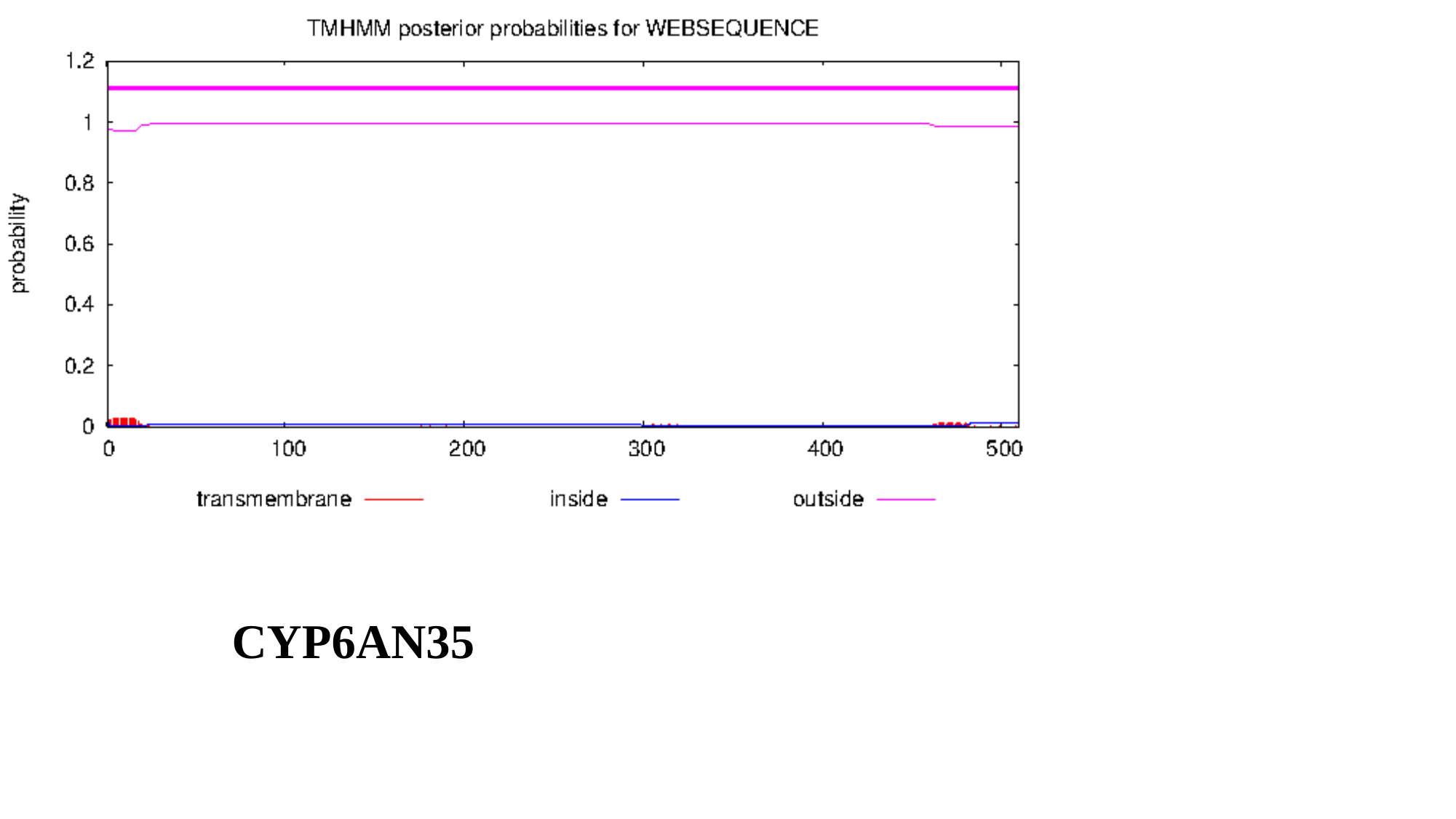

CYP6AN35

## Slide 9
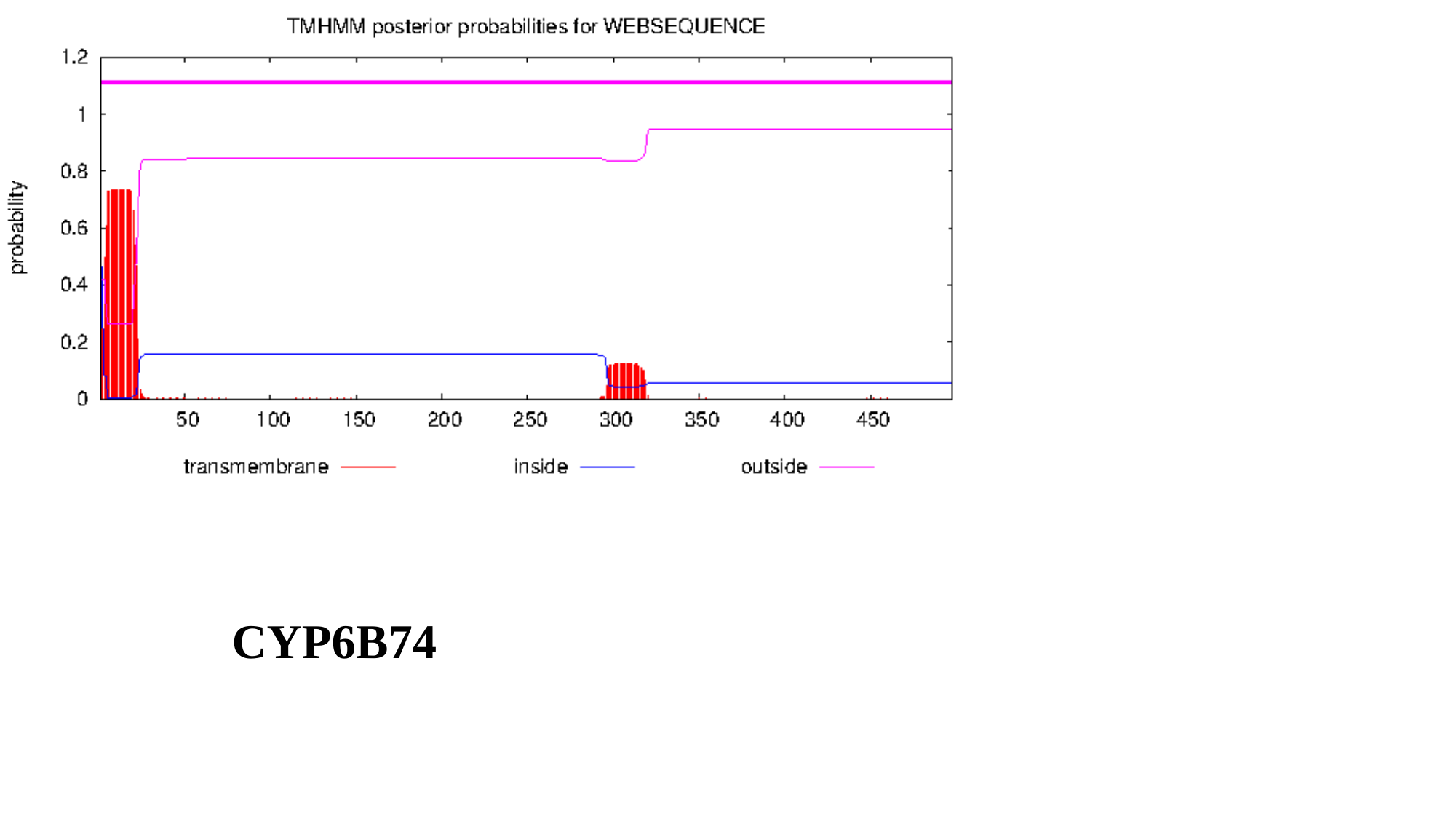

CYP6B74

## Slide 10
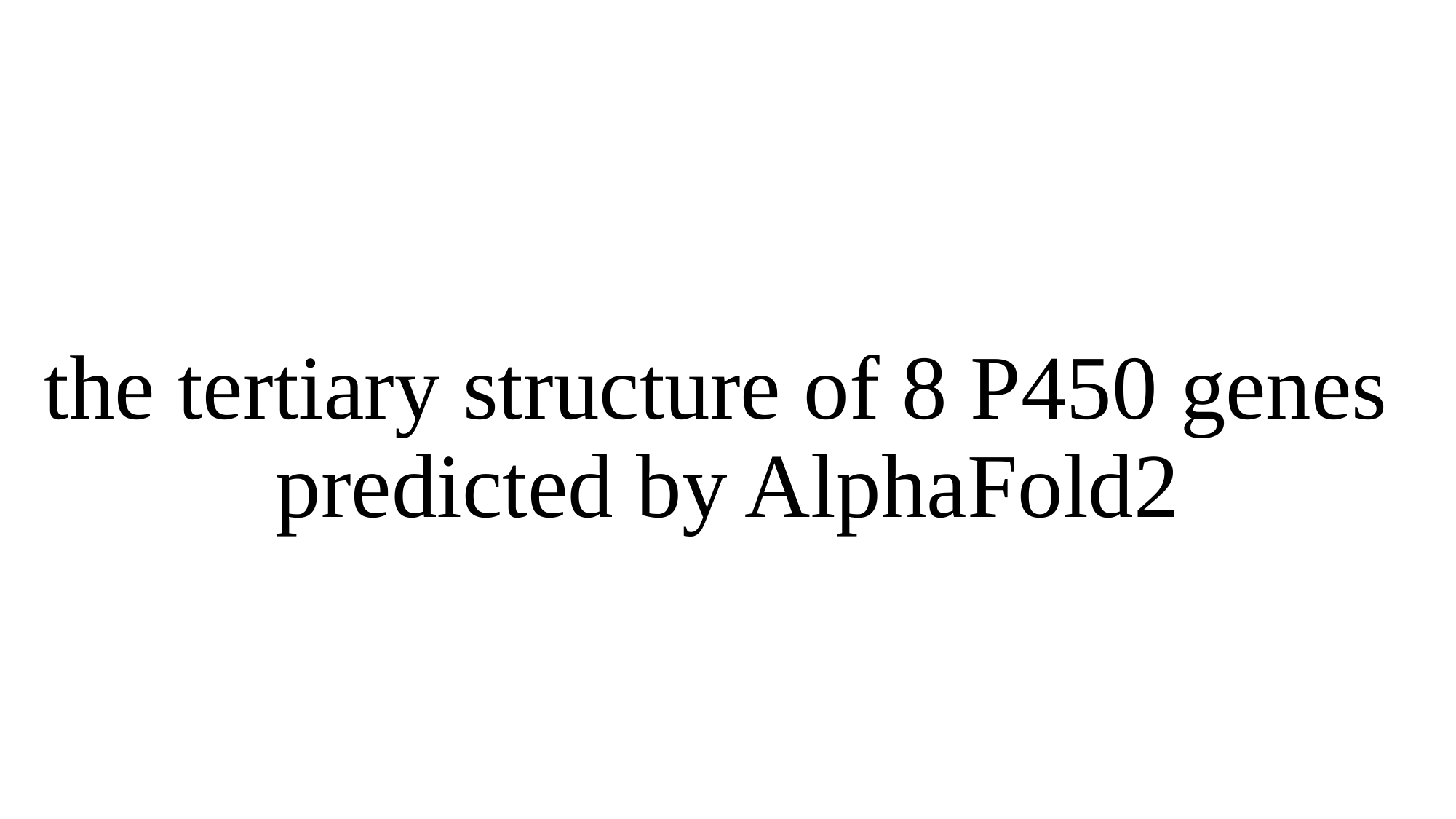

the tertiary structure of 8 P450 genes
predicted by AlphaFold2

## Slide 11
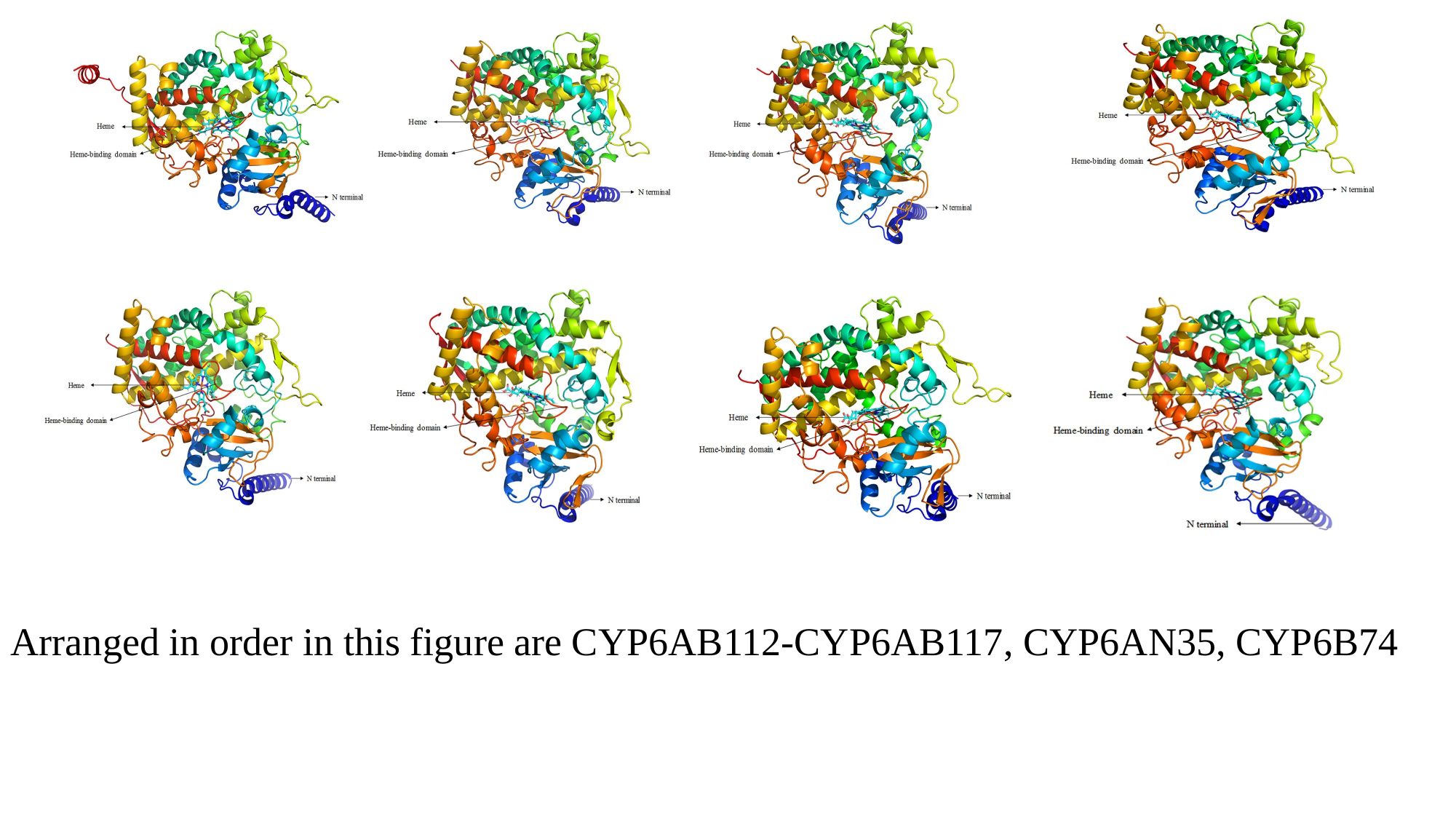

Arranged in order in this figure are CYP6AB112-CYP6AB117, CYP6AN35, CYP6B74
