# Supplementary material for: Identification and Characterization of CYP6 Family Genes from the Oriental Fruit Moth (Grapholita molesta) and Their Responses to Insecticides
Source: Insects. 2022 Mar 17;13(3):300. doi: 10.3390/insects13030300 (PMC8953268; doi:10.3390/insects13030300)
Supplement: Supplementary file 1 [file insects-13-00300-s001.zip › Supplemental Material.pdf]

Table S1 Quantitative PCR primers

Primer name	Sequence from 5′ to 3′	Amplification product length
CYP6B74	F: GAGCTAACTTGTTCCACGCC	225
	R: CGAAGACACAGGCCGTTATG	
CYP6AB112	F: TACCAGCACTCGGAAACCAT	214
	R: TCGCTTCCAAGGTGTGATCT	
CYP6AB113	F: TTTGGTTTCCTTACACGCCG	170
	R: TCATCCTCAGGGTCGAATCG	
CYP6AB114	F: TCGGCCGGAGAGATTTAGTC	206
	R: ACCCGACTTTGGCTCAACTA	
CYP6AB115	F: TTCAGATCAACACGTCCGGA	170
	R: AACAACCGCCACAAATCTCC	
CYP6AB116	F: GATGACACGTCCACATTCCG	252
	R: AAAGCGACCGTATGCCTTTC	
CYP6AB117	F: GCTGCTAAACCAACACACGA	209
	R: GCGTAGAAAATGTTGGGCCA	
CYP6AN35	F: ACCAGCCCTCATCATCAGAG	152
	R: GCAGCTTCCAAGTATCACCG	
β-actin	F: CTTTCACCACCACCGCTG	222
	R: CGCAAGATTCCATACCCA	

Table S2 Best BLASTp matches for *G. molesta* P450s

Gene name	ORF length	Best BLASTp match				
		Name	Accession number	Species	E-value	Percent identity
CYP6B74	1497	cytochrome P450 6B45	ADE05578.1	*Manduca sexta*	0	54.92
CYP6AB112	1593	cytochrome P450 6B2c	AXP17138.1	*Cydia pomonella*	0	87.61
CYP6AB113	1530	escape protein 2	AXP17154.1	*Cydia pomonella*	0	75.50
CYP6AB114	1536	cytochrome p450 *CYP6AB14*	ASO97998.1	*Vanessa tameamea*	0	69.45
CYP6AB115	1536	*CYP6AB47*	AHW57301.1	*Chilo suppressalis*	0	69.03
CYP6AB116	1530	*CYP6AB7*	ABL60877.1	*Depressaria pastinacella*	0	63.53
CYP6AB117	1551	Cytochrome P450 6B2b	AXP17131.1	*Cydia pomonella*	0	80.57
CYP6AN35	1533	Cytochrome P450 *CYP6AN19*	ASX93976.1	*Zygaena filipendulae*	0	58.62

Table S3 Physicochemical properties of *G. molesta* P450s

Gene name	CYP6B74	CYP6AB112	CYP6AB113	CYP6AB114	CYP6AB115	CYP6AB116	CYP6AB117	CYP6AN35
Number of AA	498	530	509	511	511	509	516	510
Molecular weight	58047.2	60754.95	58261.07	58496.04	58267.78	57365.67	59752.42	58195.37
Theoretical pI	8.84	8.35	6.55	8.82	8.57	8.77	7.61	8.82
Total number of negatively charged residues (Asp + Glu)	61	67	63	64	64	54	67	63
Total number of positively charged residues (Arg + Lys)	69	70	61	70	68	61	68	71
Instability index	36.62	38.77	37.22	35.79	34.96	39.57	36.35	34.77
Aliphatic index	84.56	85.36	89.02	90.84	89.55	94.09	90.41	85.47
Grand average of hydropathicity	-0.319	-0.348	-0.229	-0.166	-0.167	-0.075	-0.156	-0.258
Formula	$C_{2636}H_{4096}N_{690}O_{737}S_{25}$	$C_{2746}H_{4287}N_{741}O_{777}S_{20}$	$C_{2631}H_{4118}N_{708}O_{749}S_{19}$	$C_{2666}H_{4184}N_{698}O_{738}S_{21}$	$C_{2649}H_{4164}N_{692}O_{739}S_{23}$	$C_{2601}H_{4097}N_{693}O_{724}S_{22}$	$C_{2749}H_{4261}N_{695}O_{756}S_{19}$	$C_{2633}H_{4132}N_{696}O_{745}S_{23}$

Table S4 Signal peptides of *G. molesta* P450s

Gene name	Max C	Max Y	Max S	Mean S	Mean D	Cleavage site	Signal peptide
CYP6B74	18 (0.627)	18 (0.775)	11 (0.984）	1-17 (0.953）	1-17 (0.872）	17-18	YES
CYP6AB112	23 (0.214)	23 (0.228)	12 (0.431)	1-22 (0.253)	1-22 (0.238)	-	NO
CYP6AB113	20 (0.253)	20 (0.296)	12 (0.461)	1-19 (0.336)	1-19 (0.312)	-	NO
CYP6AB114	22 (0.165)	22 (0.224)	13 (0.548)	1-21 (0.319)	1-21 (0.262)	-	NO
CYP6AB115	22 (0.199)	13 (0.242)	1 (0.639)	1-12 (0.401)	1-12 (0.306)	-	NO
CYP6AB116	26 (0.146)	26 (0.340)	13 (0.928)	1-25 (0.761)	1-25 (0.567)	25-26	YES
CYP6AB117	33 (0.183)	23 (0.333)	15 (0.863)	1-22 (0.728)	1-22 (0.547)	22-23	YES
CYP6AN35	21 (0.173)	21 (0.336)	6 (0.737)	1-20 (0.647)	1-20 (0.504)	20-21	YES

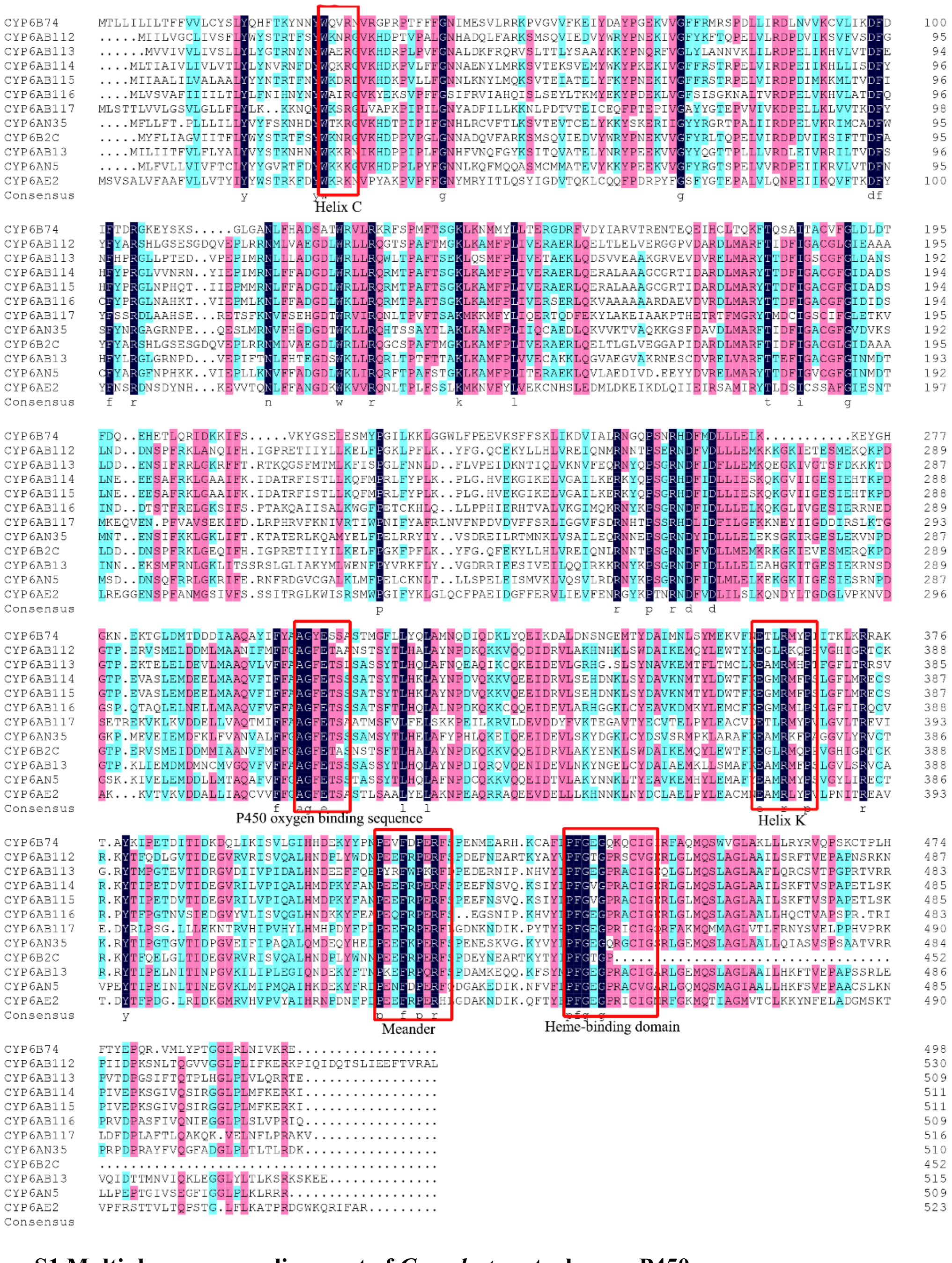

Figure S1 Multiple-sequence alignment of *G. molesta* cytochrome P450s.

Note: The following species (with GenBank accession numbers) were examined: *C. pomonella* (AXP17138.1 *CYP6B2C*), *M. sexta* (ADE05585.1 *CYP6AB13*, ADE05584.1 *CYP6AN5*), *Bombyx mori* (NP_001077080.1 CYP6AE2). The newly identified 8 genes belong to the cytochrome P450 family. The conserved region of cytochrome P450s is marked, such as 'WXXXR', 'EXXRXXP', 'PXXFXPXXF', 'FXXGXXXCXG' and 'A/GGXD/ETT/S'.
